# Activity of the neuroendocrine axes in patients with polymyalgia rheumatica before and after TNF-α blocking etanercept treatment

**DOI:** 10.1186/ar4017

**Published:** 2012-08-15

**Authors:** Frederik Flindt Kreiner, Henrik Galbo

**Affiliations:** 1 Institute for Inflammation Research, Department of Rheumatology, Rigshospitalet, Copenhagen University Hospital, Blegdamsvej 9, DK-2100 Copenhagen, Denmark

## Abstract

**Introduction:**

In this study, we evaluated the activity of the neuroendocrine axes in patients with polymyalgia rheumatica (PMR) before and after tumor necrosis factor (TNF)-α-blocking etanercept treatment, which previously has been shown to reduce interleukin 6 (IL-6) and C-reactive protein (CRP) markedly in PMR.

**Methods:**

Plasma samples were collected from 10 glucocorticoid-naïve patients with PMR and 10 matched controls before and after etanercept treatment (25 mg biweekly for 2 weeks). The primary end points were pre- and posttreatment levels of adrenocorticotropic hormone (ACTH), cortisol, adrenaline, thyroid-stimulating hormone (TSH), follicle-stimulating hormone (FSH), prolactin, and insulin-like growth factor 1 (IGF-1).

**Results:**

Before TNF-α-blocking treatment, plasma TNF-α, ACTH, and cortisol levels were higher in patients versus controls (*P *< 0.05 and *P *< 0.001, respectively); during TNF-α blockade in patients, levels of both hormones decreased (*P *< 0.05 and *P *< 0.01, respectively), whereas levels in controls increased (*P *< 0.05), abolishing the pretreatment differences. Pretreatment adrenaline levels were more than twice as high in patients than in controls (*P *< 0.01); after treatment in patients, levels had decreased (*P *< 0.05) but remained higher versus controls (*P *< 0.05). Levels of the other hormones never differed significantly between groups (*P *> 0.05).

**Conclusions:**

In PMR, TNF-α may increase the activities of the hypothalamic-pituitary-adrenal and the hypothalamic-sympthoadrenomedullary axes. Secretion of TSH, FSH, prolactin, and IGF-1 is not clearly changed in PMR.

**Trial registration:**

ClinicalTrials.gov (NCT00524381).

## Introduction

Polymyalgia rheumatica (PMR) is the most common chronic inflammatory rheumatic disease in the elderly [1]. Clinical symptoms include tenderness, aching, and stiffness in proximal parts of the limbs [1,2]. Based on histologic and imaging evidence, PMR is commonly seen as reflecting inflammation in synovial structures (that is, joints, bursae, and tendon sheaths). Recently, however, we have found that primary muscle pathology is probably also involved in the pathophysiology of PMR [3,4]. Correspondingly, PMR symptoms may be seen in the absence of imaging evidence of synovial inflammation, and, conversely, such evidence may be present without PMR symptoms or remain after such symptoms have disappeared [5,6]. Paraclinically, PMR is associated with increased erythrocyte sedimentation rate (ESR), as well as increased blood levels of C-reactive protein (CRP) and of proinflammatory cytokines [7] (for example, interleukin (IL) 6 [1,3,8-11] and, in some studies, tumor necrosis factor (TNF)-α [3,8,11-15]).

Based on TNF-α-blocking studies, it has been concluded that in rheumatoid arthritis (RA) and other chronic inflammatory diseases, such as ankylosing spondylitis, psoriasis, and Crohn disease, increased concentrations of TNF-α are associated with neuroendocrine changes, including dysfunctional hypothalamic-pituitary-adrenal (HPA), hypothalamic-pituitary-gonadal (HPG), hypothalamus-pituitary-liver-muscle, and hypothalamic-autonomic nerve system (HANS) axes (reviewed in [16]).

In PMR, knowledge of the potential pathophysiologic involvement of (neuro)endocrine dysfunction in general and the impact of TNF-α blocking in particular is modest. We previously showed that PMR is associated with decreased insulin sensitivity [8]. Reports of normal basal thyroid-stimulating hormone (TSH) [17] and prolactin concentrations [17,18] in plasma, normal [17] or elevated [2] 17-hydroxyprogesterone (17-OHP) and reduced dehydroepiandrosterone [19] responses to adrenocorticotropic hormone (ACTH) [2], and normal [17,20] or low [19] basal levels of androstenedione are also available. However, most focus has been on cortisol secretion. Challenging the immediate expectation (that is, that the HPA axis would be enhanced, the predominant view has been that, in contrast, it is impaired in PMR [2,19-21]. This view is in line with the facts that PMR symptoms are reminiscent of adrenocortical insufficiency and the steroid-withdrawal syndrome [2,16,22], and that symptoms are ameliorated by exogenous glucocorticoid administration. However, basal plasma concentrations of ACTH and cortisol have been found to be normal, and not reduced, in PMR [2,11,17,19,20,23]. Still, although not reduced, ACTH and cortisol secretion can be regarded as low relative to inflammation status [2,19-21]. This view is based on studies showing increased levels of ACTH and cortisol in response to IL-6 injection in humans [12,14,15].

In a recent randomized controlled trial, we explored the clinical effect of TNF-α blockade in glucocorticoid-naïve PMR patients [13]. However, the administration of a TNF-α blocker can also be used to elucidate the role of TNF-α in pathophysiologic mechanisms. So, to extend further the understanding of the role of the autonomic (neuro)endocrine system in the pathophysiology of PMR and with particular emphasis on the involvement of TNF-α in endocrine activity, we have now, in a subset of patients and healthy control subjects from that trial, measured the plasma levels of various hormones reflecting the activity of all of the anterior pituitary hormone axes as well as of the sympathoadrenal system before and after 14 days of TNF-α-blocking treatment with etanercept. To our knowledge, this is the first study of the impact of TNF-α blockade on the endocrinology of PMR.

## Materials and methods

### Subjects

Subject characteristics and trial protocols were previously described in detail [13]. In brief, 20 glucocorticoid-naïve patients with untreated, newly diagnosed PMR according to the Chuang criteria [24], as well as 20 healthy control subjects, were randomized in a 1:1 ratio to treatment for 14 days with the soluble Fc-coupled TNF-α receptor etanercept (Enbrel; Wyeth Pharmaceuticals New Lane, Hampshire, UK) or placebo (saline). Exclusion criteria were as follows: prior or current use of glucocorticoids or other immunosuppressive drugs; signs of giant cell arteritis, including cranial symptoms of vasculitis (headache, visual disturbances, jaw claudication, abnormal pulsation of wall of the temporal artery, scalp tenderness); infections with systemic impact; hepatitis B or C infection; positive tuberculosis-screening tests (thorax x-ray imaging, Mantoux skin test, and Quantiferon tuberculosis blood test); positive blood or urine culture; uncontrolled diabetes mellitus; uncontrolled hypertension; severe heart failure (New York Heart Association class 3 and 4); other inflammatory diseases than PMR; cancer in the past 5 years; neuromuscular disease; thyroid disease; or disturbance of calcium homeostasis. Control subjects fulfilled the same exclusion criteria as patients and were matched according to sex, age, and body mass index (BMI).

All scientific and technical personnel were blinded to group assignment. In the present investigation, only the subjects treated with etanercept were included (10 patients with PMR and 10 control subjects).

Concurrent use of glucocorticoids and nonsteroidal antiinflammatory drugs was not allowed; other usual medications were allowed, but subjects were not included if treated with drugs with potential impact on study end points [13]. Until the day of experiments, for pain control, the centrally active opioid-like analgesic tramadol (Mandolgin; Sandoz A/S, Odense, Denmark) was administered according to needs.

The study was approved by the Danish Medicines Agency (approval number 2612-3497) and The Ethical Committee of the Capital Region (approval number H-D-2007-0040), and it was entered in the EUdract (number 2007-003009) and clinicaltrials.gov (NCT00524381) databases. Before inclusion in the study, all participants signed a written informed consent.

### Protocol

Subjects were examined before and after treatment with etanercept for 14 days (biweekly subcutaneous injections with 25 mg Enbrel). In the morning before each examination, subjects were allowed to take their prescribed medications but abstained from tramadol. On both examinations, after an overnight fast, subjects arrived at 08:00 hours to the laboratory by taxi. Between 08:00 and 09:00, after at least 15 min of rest in a chair, blood samples were drawn from a cannulated forearm vein. In addition, a clinical examination of joint mobility as well as muscle function and tenderness was performed. Finally, remaining data for calculation of the PMR activity score (PMR-AS) were collected (see Study end points).

### Study end points

In the present study, as the primary end points, we measured plasma levels of ACTH, cortisol, adrenalin, follicle-stimulating hormone (FSH), prolactin, TSH, and insulin-like growth factor 1 (IGF-1), and adrenalin before and after etanercept treatment. In addition, PMR-AS was calculated from plasma CRP levels, the duration of morning stiffness, the ability to raise arms, as well as physician's global assessment and subject's assessment of pain (visual analogue scales) [13].

### Blood samples

Blood samples were drawn in stock EDTA Vacutainers with added aprotinin (Trasylol; proteolysis inhibitor); plasma was harvested by centrifugation at 1,200 rpm and 4°C for 15 minutes and frozen at -80°C until analysis within 6 months. In separate Vacutainers, blood was drawn for immediate ESR and CRP measurements.

### Analytic methods

Plasma levels were determined by using enzyme-linked immunosorbent assay (ELISA) kits; specific kits were (detection limit): ACTH, ALPCO 21-ACTHU-E01 (0.22 pg/ml); cortisol, Labor Diagnostic Nord GmbH (LDN) MS E-5000 (0.4 μg/ml); adrenalin, LDN BA E-5100 (3.9 pg/ml); FSH, LDN FR E-2400 (1 IU/L); prolactin, LDN FR E-2900 (10 μIU/ml); TSH, LDN TF E-3000 (0.05 μIU/ml); and IGF-1, Mediagnost E20 (0.09 ng/ml). Details on measurement of blood ESR and CRP as well as of plasma IL-6 and TNF-α were given in reference [13]. All analyses of a given hormone were carried out in duplicate and in a single assay.

### Statistics

Statistical analysis was performed by using SPSS 20.0 for Mac (SPSS Inc., Chicago, IL, USA). Comparisons within patients (paired data) and comparisons between patients and controls (unpaired data) were performed by using the Wilcoxon signed-rank test and the Mann-Whitney *U *test, respectively (two-way analysis of variance tests returned the same significances). *P *values < 0.05 in two-tailed testing were considered significant. Data in figures are given as medians and interquartile ranges. To allow comparison with previous reports, most of these data are also given in the Results section as mean ± SEM.

## Results

Baseline anthropometrics, including age, BMI, and blood pressure, did not differ between groups; ESR and CRP as well as IL-6 and TNF-α were significantly higher in patients with PMR versus control subjects (Table 1) [13]. As previously reported [13], before treatment, PMR disease activity, as measured by the PMR-AS, was significantly higher in patients with PMR than in controls subjects (*P *< 0.0001 to 0.001); in patients after etanercept treatment, PMR-AS had decreased significantly by 24% (95% confidence interval, 12% to 33%; *P *= 0.011) but remained markedly higher than in controls (*P *< 0.0001 to 0.001). In response to etanercept treatment in patients, ESR, CRP, and IL-6 decreased but remained significantly higher than in controls (Table 1). TNF-α levels increased in both patients and controls because of accumulation of etanercept-TNF-α complexes [13]. After 14 days of etanercept therapy, patients commenced prednisolone treatment (20 mg/day), which within a week induced complete clinical remission and normalized ESR and CRP levels, supporting the PMR diagnosis (data not shown).

**Table 1:** Characteristics of patients and control subjects before and after etanercept treatment

	Patients (*n *= 10)		Control subjects (*n *= 10)	
	
	Before	After	Before	After
Sex (female/male)	6/4	-	9/1	-
Age (years)	72.6 ± 2.6	-	69.6 ± 1.2	-
Onset-to-study duration (range; months)	8.7 ± 2.2 (2-20)	-	-	-
BMI (kg/m^2^)	23.6 ± 3.4	-	22.5 ± 0.9	-
Blood pressure (systolic/diastolic, mm Hg)	160 ± 5/87 ± 4	-	148 ± 5/85 ± 3	-
Smokers (*n*)	2	-	2	-
Hypertension (*n*)	6	-	5	-
Hypercholesterolemia (*n*)	3	-	2	-
ESR (mm/h)	71.1 ± 8.4	56.6 ± 12.6^a^	11.0 ± 3.1^e^	9.4 ± 2.8^e^
CRP (mg/dl)	7.5 ± 1.9	3.5 ± 1.2^b^	1.1 ± 0.1^e^	< 1.0^e ^
IL-6 (pg/ml)	76.4 ± 31.3	22.1 ± 4.4^c^	4.1 ± 1.6^e^	4.7 ± 1.8^e^
TNF-α (pg/ml)	9.1 ± 1.6	16.9 ± 2.6^d^	3.8 ± 0.5^e^	16.4 ± 3.2^d^

Data are expressed as mean ± SEM. ^a^Values are high due to accumulation of TNF-α/etanercept complexes; for details, please see reference [13]. ^b^*P *= 0.049 versus untreated patients, ^c^*P *= 0.007 versus untreated patients. ^d^*P *= 0.032 versus untreated patients. ^e^*P *< 0.01 versus patients.

### ACTH and cortisol

**Figure 1:**
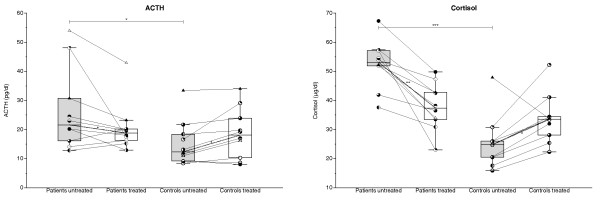
**Plasma levels of adrenocorticotropic hormone (ACTH) and cortisol before (gray bars) and after (white bars) 14 days of TNF-α-blocking treatment with etanercept in glucocorticoid-naïve patients with polymyalgia rheumatica (PMR) and in non-PMR control subjects**. Box plots show median values and 25% to 75% interquartile ranges. Symbols connected by full lines represent values for individual subjects. Dashed lines represent tendencies between medians of paired data. *P < 0.05; **P < 0.01; ***P < 0.001.

Before TNF-α-blocking treatment, levels of ACTH and cortisol (Figure 1) were significantly higher (*P *= 0.04 and *P *< 0.0001, respectively) in patients (26.4 ± 4.5 pg/ml and 52.6 ± 2.6 μg/dl, respectively) than in controls (15.3 ± 2.4 pg/ml and 25.5 ± 2.9 μg/dl, respectively). In patients with TNF-α blockade, ACTH (20.6 ± 2.6; *P *= 0.045) and cortisol (37.9 ± 2.5; *P *= 0.005) decreased. In contrast, in control subjects, levels of both ACTH (18.6 ± 2.7; *P *= 0.04) and cortisol (33.6 ± 2.6; *P *= 0.03) increased with TNF-α blockade. Consequently, postblockade levels of neither cortisol nor ACTH differed between patients and controls (*P *> 0.05).

### Ratios between ACTH and cortisol, respectively, and CRP or IL-6

**Figure 2:**
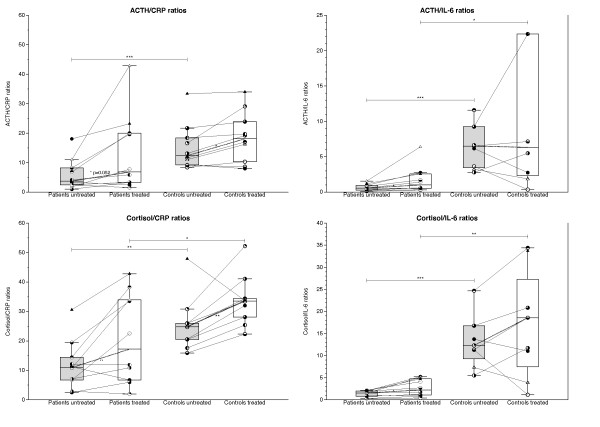
**Ratios between plasma levels of ACTH and cortisol, respectively, and IL-6 or CRP before (gray bars) and after (white bars) 14 days of TNF-α-blocking treatment with etanercept in glucocorticoid-naïve patients with polymyalgia rheumatica (PMR) and in non-PMR control subjects**. Box plots show median values and 25% to 75% interquartile ranges. Symbols connected by full lines represent values for individual subjects. Dashed lines represent tendencies between medians of paired data. *P < 0.05; **P < 0.01; ***P < 0.001.

Before TNF-α-blocking treatment, ACTH/CRP (patients 5.9 ± 1.7 versus controls 15.3 ± 2.4, *P *= 0.006), cortisol/CRP (patients 11.8 ± 2.7 versus controls 25.5 ± 2.9; *P *= 0.002), ACTH/IL-6 (patients 0.7 ± 0.1 versus controls 9.3 ± 2.8; *P *= 0.007), and cortisol/IL-6 (patients 1.4 ± 0.2 versus controls 16.8 ± 3.9; *P *= 0.0008) ratios were significantly lower in patients than in controls (Figure 2). With treatment, these ratios significantly increased in patients (ACTH/CRP 12.9 ± 4.2; *P *= 0.049; cortisol/CRP 20.9 ± 4.8; *P *= 0.02; ACTH/IL-6 1.7 ± 0.6; *P *= 0.046; cortisol/IL-6 2.7 ± 0.6; *P *= 0.01) but remained lower (*P *< 0.05) than in controls ACTH/CRP 18.6 ± 2.7, cortisol/CRP 33.6 ± 2.6, ACTH/IL-6 22.4 ± 8.6, cortisol/IL-6 20.8 ± 2.9) (Figure 2).

### Adrenalin

**Figure 3:**
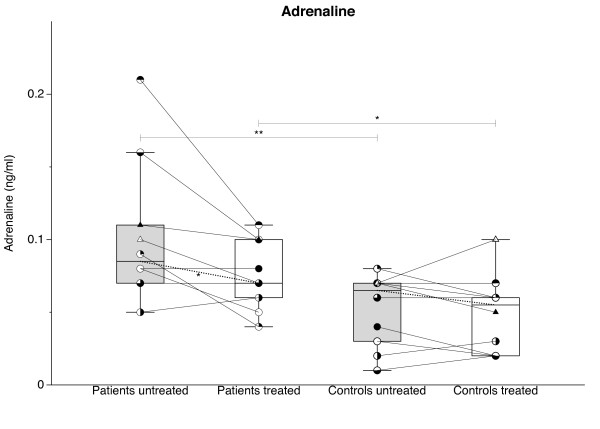
**Plasma levels of adrenalin before (gray bars) and after (white bars) 14 days of TNF-α[blocking treatment with etanercept in glucocorticoid-naïve patients with polymyalgia rheumatica (PMR) and in non-PMR control subjects**. Box plots show median values and 25% to 75% interquartile ranges. Symbols connected by full lines represent values for individual subjects. Dashed lines represent tendencies between medians of paired data. *P < 0.05; **P < 0.01.

Before TNF-α-blocking treatment, levels of adrenalin (Figure 3) were considerably higher in patients compared with controls (patients 0.11 ± 0.02 versus controls 0.051 ± 0.007 ng/ml; *P *= 0.007); with TNF-α blockade, levels in patients (0.077 ± 0.006; *P *= 0.03) but not in controls (0.046 ± 0.009; *P *> 0.05) decreased, but levels in patients remained higher (*P *= 0.03) than in controls.

### TSH, FSH, prolactin, and IGF-1

**Figure 4:**
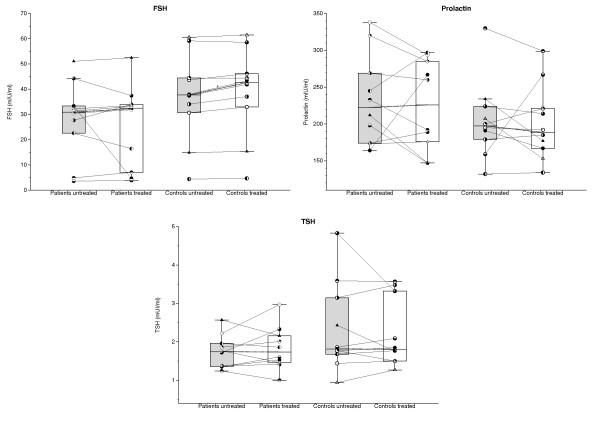
**Plasma levels of follicle-stimulating hormone (FSH), prolactin, and thyroid-stimulating hormone (TSH) before (gray bars) and after (white bars) 14 days of TNF-α-blocking treatment with etanercept in glucocorticoid-naïve patients with polymyalgia rheumatica (PMR) and in non-PMR control subjects**. Box plots show median values and 25% to 75% interquartile ranges. Symbols connected by full lines represent values for individual subjects. Dashed lines represent tendencies between medians of paired data. *P < 0.05.

**Figure 5:**
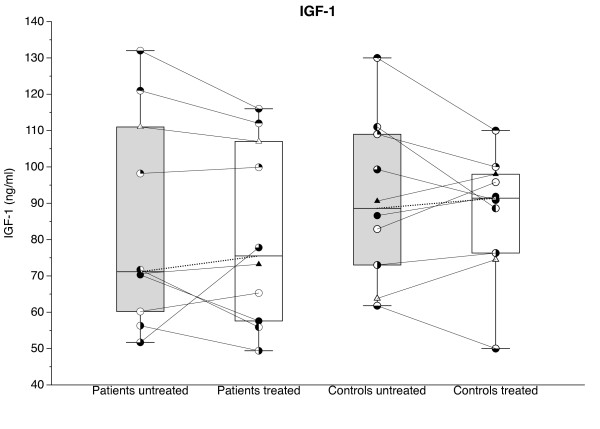
**Plasma levels of insulin-like growth factor 1 (IGF-1) before (gray bars) and after (white bars) 14 days of TNF-α-blocking treatment with etanercept in glucocorticoid-naïve patients with polymyalgia rheumatica (PMR) and in non-PMR control subjects**. Box plots show median values and 25% to 75% interquartile ranges. Symbols connected by full lines represent values for individual subjects. Dashed lines represent tendencies between medians of paired data.

Levels of TSH, FSH, and prolactin (Figure 4) as well as of IGF-1 (Figure 5) did not differ significantly between groups before or after TNF-α blockade. However, levels of TSH, FSH, and IGF-1 tended (*P *< 0.1) to be lower in patients than in controls both before and after treatment, whereas mean levels of prolactin were always higher in the patients (*P *< 0.1).

## Discussion

The major new finding of the present study is that in glucocorticoid-naïve patients with PMR, the plasma concentrations of ACTH and cortisol, as well as of adrenalin, may be increased compared with findings in healthy controls. Furthermore, these differences were abolished and reduced, respectively, during 14 days of TNF-α-blocking treatment with etanercept. Levels of TSH, FSH, and IGF-1 tended to be lower, and those of prolactin, higher, in patients than in controls before as well as after treatment, but differences did not achieve statistical significance, and concentrations were not influenced by etanercept treatment.

### ACTH and cortisol

In previous studies, basal concentrations of ACTH and cortisol in plasma were found to be normal [1,2,11,17,19,20]. Furthermore, supporting the view that the HPA axis is intact in PMR, stimulation with corticotropin-releasing hormone (CRH) produced normal ACTH and cortisol responses [1,2], whereas stimulation with ACTH has been found to elicit either normal [2-4], increased [5,6,19], or slightly, if at all, reduced [7,17] cortisol responses. Still, it has been proposed that in PMR, the HPA axis is inhibited and that this may contribute to the symptoms [2,19]. Although not reduced in absolute terms in PMR, cortisol secretion may be relatively impaired and inadequate relative to inflammation [25]. This is so, because plasma cortisol levels may be lower than expected, because concentrations of various cytokines (for example, IL-6) are increased [1,3,8-11], and because in non-PMR patients, injection with IL-6 has been shown to increase ACTH and cortisol levels in plasma [3,8,11-15]. Emphasis has been put on ratios between concentrations of hormones and inflammatory variables (for example, cortisol/CRP and ACTH/CRP) [17,26]. In line with a previous study in PMR [17], we also found that before treatment, these ratios as well as cortisol/IL-6 and ACTH/IL-6 ratios were significantly lower in patients than in controls (Figure 2).

Nonetheless, an important addition of the present study to existing evidence is that in untreated PMR, increased basal levels of ACTH and cortisol in plasma may also be found (Figure 1), indicating activation of the HPA axis, as seen in other stress conditions [27]. Whether, in the present study, the activation of the HPA axis was less than expected (from the concomitant increased levels of inflammatory cytokines) is difficult to tell. For instance, before etanercept treatment, cortisol concentrations were 110% higher in PMR patients compared with controls, and this difference corresponds with what one would expect from the IL-6 plasma concentration difference between the two groups and the relation between cortisol and IL-6 concentrations determined by IL-6 injections in healthy subjects [15,25]. However, in PMR, IL-6 is most likely only one of the variables involved in a complex overall setting of the hypothalamus stimulation from inflammatory and other factors, and dose-effect studies intending to mimic the simultaneous influence of all changes in the internal milieu seen in inflammatory conditions have not been performed. That ratios between hormones and cytokines are difficult to interpret is illustrated by the fact that, even though serum cortisol and IL-6 concentrations were linearly related in the quoted study of IL-6 injections [15], calculated ratios between cortisol and IL-6 decreases with increasing IL-6 concentrations, because the regression line did not originate in the (0,0).

One possible explanation for the differences in ACTH and cortisol levels between our study and previous studies is that before treatment with etanercept, disease activity in our patients was higher than that of previously studied patients. Thus, mean ESR and CRP levels in our study before treatment were 71 mm/h and 7.5 mg/dl, respectively, whereas mean values in previous investigations, which did not find increased ACTH and cortisol levels, were 34 to 72 mm/h (range) and 0.7-4.9 mg/dl, respectively [2,11,17,21,23]. In the present study, after TNF-α-blocking treatment with etanercept, ESR and CRP had declined to values (means, 57 mm/h and 3.5 mg/dl, respectively) comparable to the mean values in the previous investigations, and this was accompanied by some, albeit modest, clinical improvement [13]. So, the normalization of ACTH and cortisol concentrations during TNF-α blocking with etanercept was associated with a reduction in disease activity.

Another factor that might explain the differences in HPA activity between studies could be that in previous studies, disease onset-to-study duration (published durations, weeks ± SD: 11.2 ± 8 [11]; 19.2 ± 10.4 [17]; 10.4 ± 0.8 [2]; 30.7 ± 24 [20]; and 10.8 ± 10 [23]) was longer compared with the present study (mean 8.7 ± 12.5 weeks, Table 1), allowing, in the former studies, a spontaneous reduction in HPA axis activity as seen in critical illness [28] or adaptation of the hypothalamus to, for example, increased stimulation with inflammatory cytokines [25]. An increase and subsequent decrease of ACTH and cortisol has been proposed to be evolutionarily advantageous, for example, in severe systemic infection [25]. Our new finding of enhanced HPA-axis activity adds to existing evidence by indicating that such a time course exists in PMR.

The mechanism for the depicted decline in HPA activity is unclear [25]. A downregulation of hypothalamic activity in response to maintained stimulation by cytokines has been proposed [25]. Compatible with this idea, ACTH responses were diminished from the beginning to the end of studies of patients with cancer injected daily with IL-6 for 7 or 21 days [12,14]. However, cortisol responses were not reduced, and the authors explained the diminished ACTH responses to IL-6 by feedback inhibition from cortisol rather than reflecting hypothalamic adaptation to IL-6 [12,14].

The second major finding of the present study is that TNF-α is probably involved in the stimulation of the HPA axis in PMR. This is so because we found that the stimulation was abolished during 14 days of TNF-α-blocking etanercept treatment (Figure 1). Supporting that, in the patients, the effect of etanercept did in fact reflect TNF-α blockade and was not unspecific, in the control subjects, the effect of the agent on ACTH and cortisol was opposite that seen in the patients (Figure 1). At variance with the present findings, based on a study of RA patients treated with anti-TNF-α antibody, it was proposed that prolonged elevation of serum TNF-α inhibits ACTH secretion at the hypothalamic or pituitary level [29]. However, in contrast to this suggestion, mean ACTH and cortisol levels apparently decreased during the initial 2 weeks of treatment and were, on average, not altered during 16 weeks of observation [29]. Correspondingly, in another study, plasma cortisol concentrations increased during 12 weeks of TNF-α blockade in RA patients with initially relatively low cortisol levels, but cortisol levels decreased in patients with relatively high levels [30]. Overall, mean concentrations apparently did not change. It was speculated that the findings reflected the existence of two types of RA patients with remarkably different TNF-α influence on the HPA axis [30]. It may also be speculated that statistical regression toward the mean played a role for the observed differential response.

### Adrenalin

As would be expected in acute inflammation, in PMR patients in the present study, activation of the HPA axis was paralleled by an increased activity in the sympathetic nervous system, as indicated by increased adrenaline concentrations in plasma (Figure 3). Furthermore, in response to the TNF-α blockade, adrenaline secretion was ameliorated along with HPA axis activity (Figures 1 and 3). Based on measurements of heart function, it has previously been concluded that sympathetic nervous system activity is increased in RA [31]. In systemic lupus erythematosus, sympathetic outflow has been found to be increased as judged from neuropeptide Y (NPY) concentrations in serum [32]. In contrast, no increase in NPY concentration was found in patients with RA, who were not treated with glucocorticoids [32]. The effect of anti-TNF-α treatment was studied in the latter patients; NPY concentrations were not influenced by the treatment, a finding which is not surprising considering the fact that concentrations were not increased before treatment [32]. Chromogranin A (CHGA) is a less specific marker of neuroendocrine secretion, the plasma concentration of which may be increased in RA [33]. No change in CHGA levels was found in RA patients treated for 6-14 weeks with anti-TNF-α antibody [33].

### Prolactin, TSH, FSH, and IGF-1

The findings in the present study of borderline significant increase in plasma prolactin and decreases in TSH, FSH, and IGF-1 (Figures 4 and 5) are compatible with a time course of hormonal changes in PMR similar to that described for critical illness [28]. In the early phase of critical illness, anterior pituitary secretion increases, whereas in prolonged disease, secretion is gradually suppressed [28]. The normal co-variation between GH secretion and IGF-1 levels in plasma is partially disrupted, IGF-1 levels being low throughout critical illness [28].

In previous studies of PMR patients, prolactin and TSH concentrations did not differ significantly from concentrations in control subjects [17,26]; however, in accordance with our findings, concentrations tended to be increased and decreased, respectively [17,26]. In a number of other inflammatory diseases, including RA, elevated levels of prolactin have been found [34]. The HPG axis, here monitored with FSH, and the hypothalamus-pituitary-liver-muscle axis, here monitored by IFG-1, have not previously been studied in PMR, nor has the effect of TNF-α blockade on neuroendocrine activity. We found no effect of TNF-α blockade on plasma prolactin, TSH, FSH, and IGF-1 levels in PMR patients (Figures 4 and 5). This is in contrast to the findings in healthy subjects, that IL-6 injection may increase concentrations of prolactin and GH, whereas both IL-6 injection and TNF-α infusion may reduce plasma TSH [15,35]. In contrast to the latter observation, in RA patients, anti-TNF-α treatment for 28 weeks has been found to decrease TSH levels in plasma [36]. However, the decrease in TSH levels was larger in hypothyroid than in euthyroid patients, a difference that probably reflected that the effect of the TNF-α-blocking treatment on TSH secretion in part was secondary to improved thyroid gland function [36].

## Conclusions

The present study has shown that in PMR, TNF-α-dependent mechanisms may enhance the activity of the HPA axis and of the sympathoadrenal system. The evidence for changes in secretion of other pituitary hormones and IGF-1 is ambiguous, and this secretion is not influenced by TNF-α blockade.

## Abbreviations

17-OHP: 17-hydroxyprogesterone; ACTH: adrenocorticotropic hormone; CHGA: chromogranin A; CRH: corticotropin-releasing hormone; CRP: C-reactive protein; ESR: erythrocyte sedimentation rate; FSH: follicle-stimulating hormone; HANS: hypothalamic-autonomic nervous system; HPA: hypothalamic-pituitary-adrenal; HPG: hypothalamic-pituitary-gonadal; IGF-1: insulin-like growth factor 1; IL-6: interleukin 6; NPY: neuropeptide Y; PMR: polymyalgia rheumatica; PMR-AS: polymyalgia rheumatica activity score; RA: rheumatoid arthritis; SEM: standard error of the mean; TNF-α: tumor necrosis factor alpha; TSH: thyroid-stimulating hormone.

## Competing interests

The authors declare that they have no competing interests.

## Authors' contributions

FK and HG contributed equally to the planning and conduct of the trial as well as to the analysis and interpretation of study outcomes. Both authors drafted and approved the manuscript.
